# Exosomal AP000439.2 from clear cell renal cell carcinoma induces M2 macrophage polarization to promote tumor progression through activation of STAT3

**DOI:** 10.1186/s12964-022-00957-6

**Published:** 2022-09-24

**Authors:** Tianyi Shen, Suqin Miao, Yulin Zhou, Xiaoming Yi, Song Xue, Bowen Du, Chaopeng Tang, Le Qu, Dian Fu, Ruipeng Jia, Haowei He

**Affiliations:** 1 Department of Urology, Jinling Hospital, Jinling School of Clinical Medicine, Nanjing Medical University, Nanjing, 210002 Jiangsu China; 2 Department of Anesthesiology, Jinling Hospital, Nanjing University Medical School, Nanjing, 210002 Jiangsu China; 3 Department of Urology, Nanjing First Hospital, Nanjing Medical University, Nanjing, 210002 Jiangsu China

**Keywords:** lncRNA AP000439.2, Clear cell renal cell carcinoma, Macrophage, Exosomes, STAT3, NF-κB signaling pathway

## Abstract

**Background:**

Tumorigenic phenotype of M2 tumor-associated macrophages promote tumor progression in response to exosomes cues imposed by tumor cells. However, the effect and underlying mechanisms of clear cell renal cell carcinoma (ccRCC)-derived exosomes (ccRCC-exo) on instructing macrophages phenotype remains unclear.

**Methods:**

Macrophages were cocultured with ccRCC-exo and then evaluate the polarization of macrophages and migration of ccRCC cells. The effect and mechanism of lncRNA AP000439.2 overexpressed or deleted exosomes on macrophages M2 polarization were examined. Xenograft tumor mice model was used for in vivo validation.

**Results:**

The ccRCC-exo significantly activated macrophages to M2 phenotype presented by increased expression of transforming growth factor-beta (TGF-β) and interleukin 10 (IL-10) at mRNA and protein levels, and these M2 macrophages in turn facilitating the migration of ccRCC cells. LncRNA AP000439.2 was highly enriched in the ccRCC-exo. Overexpression of exosomal AP000439.2 promoted M2 macrophage polarization whereas AP000439.2-deficient exosome had the opposite effects. Nuclear-localized AP000439.2 directly interacted with signal transducer and activator of transcription 3 (STAT3) proteins and phosphorylated STAT3 in macrophages. RNA-Seq results showed overexpression of AP000439.2 activated NF-κB signaling pathway. Silencing of STAT3 suppressed overexpression of AP000439.2-induced up-regulation of TGF-β and IL-10 expression, and p65 phosphorylation. AP000439.2-deleted exosome inhibited tumor growth in vivo.

**Conclusion:**

Exosomes from ccRCC deliver AP000439.2 to promote M2 macrophage polarization via STAT3, thus enhancing ccRCC progression, indicating exosomal AP000439.2 might be a novel therapeutic target in ccRCC.

**Video Abstract**

**Supplementary Information:**

The online version contains supplementary material available at 10.1186/s12964-022-00957-6.

## Introduction

Renal cell carcinoma (RCC) is the eighth leading malignancy in the world, comprising 85% of malignant RCC and accounting for 2–3% of all malignant diseases in adults [1, 2]. Pathologically, clear cell RCC (ccRCC) accounts the majority of RCC with a frequency of 70–80% [3], characterized by compact nests of tumor cells with clear cytoplasm separated and commonly von Hippel-Landau inactivation [4, 5]. Currently, the commonly therapies, including radical nephrectomy, surgical nephrectomy or medical treatment [6], are difficult to solve the problems of relapse or resistance [7]. Therefore, for exploring specific biomarkers and therapeutic targets, it is highly necessary to understand the molecular mechanism underlying ccRCC progression.

In the tumor microenvironment (TME), tumor-associated macrophages (TAMs) were elucidated to the highly plastic and heterogeneous immune cell types, which can differentiate into a classically (M1) or alternatively (M2) activated type under different stimulation factors [8]. The M2 macrophage supposed to be tumorigenic phenotype with producing high levels of anti-inflammatory cytokines [9]. Recently, tumor cells have been suggested to secrete polarization factors, such as high-level lipid production [8] or exosome-loaded long noncoding RNAs (lncRNAs) [10], to induce M2 polarization of macrophages to support their own malignant progression. Importantly, exosomes are considered performing a significant role in the macrophages tumorigenic polarization orchestrating by tumor cells [11], but the underlying mechanism remains largely unknown.

Exosomes are nano-scale extracellular vesicles with 30–150 nm and originate from endolysosomal compartment of cells [11]. Exosomes can mediate intercellular communication by shuttling lncRNAs to recipient cells and releasing the cargoes to trigger metabolic pathways activation. For instance, exosome-mediated transfer of lncRNA H19 to induce doxorubicin resistance in breast cancer [12] or gefitinib resistance in non-small cell lung cancer [13]. Moreover, macrophages are one of the vital recipient cells of exosomes derived from tumor cells. For example, exosomes from hepatocellular carcinoma cells delivering DLX6-AS1 into macrophages trigger microRNA-15a-5p/C-X-C motif chemokine ligand 17 (CXCL17) axis resulting in M2 macrophage polarization [10]. Exosomal lncRNA breast cancer-related transcript 1 (BCRT1) induces M2 macrophage polarization by activating miR-1303/polypyrimidine tract binding protein 3 (PTBP3) axis in breast cancer [14]. However, whether exosome from ccRCC cells can polarize pro-tumorigenic M2 macrophage by shuttling lncRNAs remain largely elusive.

In the present study, we investigated the effect of ccRCC-derived exosomes on TAMs and identified the key exosomal lncRNA in this process. Moreover, transcriptome sequencing revealed changes in macrophage expression profile caused by overexpression of candidate lncRNA, as well as the strongly responsive molecules and pathways. Our study provide novel insight into the polarization mechanism of macrophage induced by ccRCC and a promising therapeutic target treatment.

## Materials and methods

### Cell culture and treatment

Human renal clear cell carcinoma cell lines 786-O and Caki-1, human normal kidney proximal tubular cell line HK-2, and human monocyte cell line THP-1 were purchased from SIBS (Shanghai, China). Caki-1 cells were cultured with McCoy’s 5A medium (PM150710, Procell, China) and other three cell lines were cultured with RPMI 1640 medium (10-040-CV, CORNING, China), these medium were contained 10% fetal bovine serum (FBS, 10099-141, GIBCO, China) and 1% Penicillin–Streptomycin (E607011, Sangon, China). In addition, THP1 cells were used to induce macrophages by treating with 100 ng/mL phorbol 12-myristate 13-acetate (PMA; Sigma, USA) for 48 h. For further M2 phenotype macrophage induction, cells were treated with 20 ng/mL IL-4 for 48 h in the presence of PMA.

### Exosome isolation

Exosomes extraction was completed as previous description [15]. Briefly, 12 h before harvested, culture medium 786-O and Caki-1 cells were replaced by fresh medium without FBS. After that, cell culture was collected by centrifuging at 300 g for 10 min. The supernatant was further centrifuged at 2000 g for 10 min to remove cellular and retained supernatant followed by filtering with a 0.2 mm filter and centrifuged again at 10,000 g for 1.5 h. The pellet was collected as exosomes and suspended in phosphate buffer saline (PBS) for using subsequently analysis. The concentration of exosomes was determined by the protein content using BCA protein assay.

### Transmission electron microscopy (TEM)

The morphology of aliquot exosomes was observed by TEM as described previously [15]. Briefly, exosmoes were fixed with 2.5% glutaraldehyde in 0.1 mol/L PBS at 4 °C overnight followed by post-fixed in PBS 1% OsO_4_ for 2 h. next, exosmoes were embedded in 10% gelatin and fixed again with glutaraldehyde at 4 °C. After that, embedded exosmoes cut into blocks and stained en bloc in uranyl acetate followed by dehydrated with ethanol. Finally, sealed with epoxy resin under standard procedures. The ultra-thin sections were observed using JEM-1200EX (JEOL, Japan).

### Nanoparticle tracking analysis (NTA)

The size and amount of exosomes were measured by NanoSight. In brief, exosomes were diluted in PBS buffer and resuspended again. ZetaView system was calibrated using polystyrene particles (110 nm). Then, exosomes sample were diluted onefold, tenfold, 100-fold, and 1000-fold with PBS buffer and loaded to sample chamber of ZetaView PMX 110 (Particle Metrix, Meerbusch, Germany) followed by the manufacturer's instructions. The same dilution of the same sample was repeated 3 times.

### Exosomes labeling

Exosomes were labeled with PKH26 (#MINI26, Sigma) according to the manufacturer’s instructions. Briefly, exosomes from the supernatant of 1.5 × 10^6^ cells were isolated and resuspended at 100 μL PBS followed by added 4 μL PKH26 dyes at 25 °C for staining 4 min. Using 1 mL FBS to terminate the reaction, and then added 200 μL ExoQuick-TC (SYSTEM BIOSCIENCES) on ice bath for 30 min. Finally, the tubes were centrifuged at 14,000 rpm for 3 min and removed supernatant to retain pellet exosomes. The final pellet containing exosomes was resuspended in 300 μL culture medium. PKH-26 labeled exosomes were co-culture with macrophages at 37 °C for 3 h followed by fixed with 4% paraformaldehyde at 25 °C to avoid light for 10 min. Finally, 4,6-diamidino-2-phenylindole (DAPI) solution was added and dyed for 10 min at 25 °C. The internalized exosomes were observed by fluorescence microscopy.

### Western blot

Proteins concentration of cells or exosomes were measured by BCA assay after treated with RIPA buffer (Sangon Biotech) containing protease inhibitors (Roche Protease Inhibitor Cocktail). Briefly, approximately 20 μg of total protein were separated with 10% SDS-PAGE and then proteins were transferred to nitrocellulose membrane (Millipore) followed by blocked with tris buffered saline Tween-20 (TBST) containing 5% non-fat dry milk. Next, blocked membrane was incubated with primary antibodies: GAPDH (60004-1-Ig, Proteintech, 1:1000), CD63 (EXOAB-CD63A-1, SBI, 1:1000), CD9 (#13403, CST), TGF-β (sc-65378, Santa, 1:500), IL-10 (bs-0698R, Bioss, 1:1000), signal transducer and activator of transcription 3 (STAT3) (#9139, CST), p-STAT3 (#9135, CST). After washed with TBST three times, membrane was incubated with horseradish peroxidase-conjugated secondary antibody (A0261, Beyotime, 1:1000, Ab6721, Abcam, 1:5000). Bands were detected by ChemiScope (CLiNX; Shanghai), and analyzed with Image J.

### qRT-PCR

Total RNA and exosome RNA were extracted with TRIzol reagent (Invitrogen, USA), and cDNA was synthesized by PrimeScript RT reagent Kit (Takara). Quantitative PCR was carried out on an ABI Q6 (Applied Biosystems Inc., USA) with the primers, as shown in Additional file 1: Table S1. The PCR protocol was: 95 °C for 10 min, followed by 45 cycles of 95 °C for 15 s, 60 °C for 60 s. Actin was used as an endogenous normalizer, and data were quantified by the 2^−ΔΔCt^ method.

### Cell migration assay

The migration ability of tumor cells was assessed by Transwell. The 0.8 μm pore size 24-well plate membranes were used in this assay (Corning, USA). In the upper chamber, 60,000 cells were seeded in culture medium (Gibco, USA). The lower chamber was filled with culture medium containing 20% FBS. After the incubated with 24 h, the filters were stained with Crystal Violet (Sangon Biotech) at 25 °C for 30 min. After sealed with neutral gum, the migration cell was counted of each filter under six different randomly fields.

### Cell transfection

To overexpression of lncRNA AP000439.2, we constructed full length of AP000439.2 into pcDNA3.1 vector (oe-AP000439.2), and the empty vector was set as a negative control (oe-NC). To interfere with lncRNA AP000439.2 expression, we designed three siRNA targeting lncRNA AP000439.2 and a control siRNA (siNC). To interfere with STAT3 expression, we also designed three siRNA targeting STAT3 and a control siRNA. The siRNAs against AP000439.2 or STAT3 were purchased from Genepharma (Suzhou, China). The sequences of siRNAs were shown in Additional file 1: Table S1. For overexpression and interference, these vector and siRNA were transfected into cells by using Lipofectamine™ 2000 (Sigma-Aldrich) according to the kit instructions. In brief, 2 μg vector or siRNA diluted to 50 μL OPTI-MEM reagent, and 5 μL Lipofectamine™ 2000 diluted to 45 μL OPTI-MEM reagent. Next, the 50 μL sample mixture was mixed with the 50 μL transfection reagent and incubated at 25 °C for 20 min. After incubation, the mixture was added to the cell samples and incubated for 6 h until subsequent experiments.

### Nuclear-cytoplasmic separation

Tumor cells were harvested and washed with ice cold PBS and then resuspended in buffer (consisted of 10 mM Tris–HCl pH 7.4, 5 mM MgCl_2_, 10 mM NaCl, 1 mM DTT, proteinase inhibitors, RNase inhibitor), and incubated on ice and mixed every 5 min for 20 min. Then, a final concentration of 0.5% NP-40 was added into the tube and mixed on ice for incubation another 1 min. Next, cells were lysed by frequent vortexing for 10 min followed by centrifuged at 3000 rpm at 4 °C for 5 min. Finally, the supernatant was collected as the cytoplasm fraction while the sediment was considered as nuclear fraction. The total RNA was extracted from these two fractions and detected by qRT-PCR.

### RNA pull-down assay

The AP000439.2 and antisense were transcription by in vitro transcription system of T7 RNA polymerase (Ambion Life, USA) to obtain AP000439.2 RNA and antisense RNA, after purified by RNeasy Plus Mini Kit (QIAGEN, USA), then, it was labeled with biotin. Labeled RNA was captured by pre-clear streptavidin magnetic beads (Life Technologies). Next, this complex was incubated with cell lysate to capture target protein at 4 °C for 50 min. The RNA–protein precipitation was washed with wash buffer and eluted. The captured protein was detected by silver staining, mass spectrometry and western blot. To determine which specific region of AP000439.2 interacts with STAT3, we truncated AP000439.2 into two parts: ^△^1 represents the region between 1 and 320 nt, ^△^2 represents the region between 321 and 726 nt. We constructed two recombinant plasmid of pcDNA3.1 (+)-AP000439.2 1-320 nt and pcDNA3.1 (+)-AP000439.2 321-726 nt. To determine which specific region of STAT3 interacts with AP000439.2, we truncated STAT3 into three parts: ^△^1 represents the region between 1 and 319 amino acid (aa), ^△^2 represents the region between 320 and 583 aa, ^△^3 represents the region between 584 and 770 aa. And then the recombinant plasmids were processed RNA pull-down assay.

### RNA immunoprecipitation (RIP) assay

The EZMagna RNA immunoprecipitation Kit (17-701; Millipore) was used to investigate whether AP000439.2 could interact potential binding proteins STAT3. 786-O cells were lysed using RIP lysis buffer to harvest cell lysates and 10% lysates were used as input. Then, left cell lysates individually incubated with magnetic beads conjugated with anti-STAT3 (SAB1406670; Millipore) antibody and control IgG (Millipore) at 4 °C for 12 h. After that, the beads were washed three times with RIP buffer. Next, added Proteinase K into tubes and incubated at 55 °C for 30 min to remove the proteins. Finally, the expression of AP000439.2 in purified RNA was detected by RT-qPCR, and U6 was served as control.

### RNA-Seq

Two groups of 786-O cell-derived exosmes containing oe-AP000439.2 or oe-NC were subjected to incubate with macrophages, and then these pretreated macrophages were performed transcriptome sequencing with triple biological repetitions. The extraction of total RNA was prepared with NEBNext Poly(A) mRNA kits (E7490, NEB) followed by treated with NEBNext Ultra II Non-Directional RNA Second Strand Synthesis Module kit (E6111, NEB) to synthesis the second strand of cDNA. Next, the cDNA libraries were constructed using Vazyme kit (ND604, Vazyme). Libraries were sequenced by Illumina Hiseq X Ten platform. Raw data were quality controlled using Fast-QC (http://www.bioinformatics.babraham.ac.uk/projects/fastqc/). Clean data were mapped to the human genome (hg38) by TopHat (version 2.1.1, http://ccb.jhu.edu/software/tophat/index.shtml). The gene expression level was calculated by Cufflinks, and when the gene expression satisfied the criteria for |log2 fold change|> 1 and false discovery rates < 0.05, it was considered a differentially expressed gene (DEG). The pathway analysis of DEG was performed by DAVID (https://david.ncifcrf.gov/).

### Tumor-bearing mouse model

5-week-old female athymic BALB/c nude mice were divided into two groups (5 mice per group): siNC-exo group and siAP000439.2-exo group. All mice with established subcutaneous ccRCC xenograft tumor model through injected 5 × 10^6^ Caki-1 cells at the flank of mice. In parallel, a dosage of 5 mg exosomes were injected into the tail vein of mice, injected every 3 days for 2 weeks. For siAP000439.2-exo group, mice were injected by AP000439.2-deleted exosome; for siNC-exo group, mice were injected by NC exosome. The growth rate of a tumor was monitored by measuring the tumor size using a digital calliper. After injected Caki-1 cells, the tumor size was continue measured every 3 days for 4 weeks. Tumor volume was calculated using the following formula: Tumor volume = length × width × width/2. The mice experiments were approved by the Animal Ethics Committee of the Jinling Hospital, Nanjing University Medical School.

### Flow cytometry assay

Tumor tissues were triturated and digested with trypsin–EDTA (11668-500, Invitrogen) to obtain single-cell suspension. Cells were cultured in ultralow attachment six-well plates (CLS3471-24EA, Corning) overnight. Appropriately 1 × 106 cells were taken used for flow cytometry and then incubated with 50 μL primary antibodies (anti-F4/80, 86007S, CST; anti-NOS2, 12-59520-82, Thermo Fisher; anti-CD163, 11-1631-82, Thermo Fisher) in an ice bath for 30 min in the dark. After incubation, cells were concentrated at 200 g for 5 min and removed supernatant. Finally, the precipitation was resuspended in PBS solution after washing and subjected to flow cytometry analysis within 1 h by FACSVerse™ (BD).

### Statistical analysis

The comparisons of means among groups were analyzed by one-way ANOVA followed by Tukey’s test. The comparisons of means between two groups were analyzed by Two-tailed Student’s t-test. A value of p < 0.05 was considered statistically significant. Statistical analyses were performed with the SPSS package.

## Results

### Identification of exosomes derived from ccRCC

**Fig. 1:**
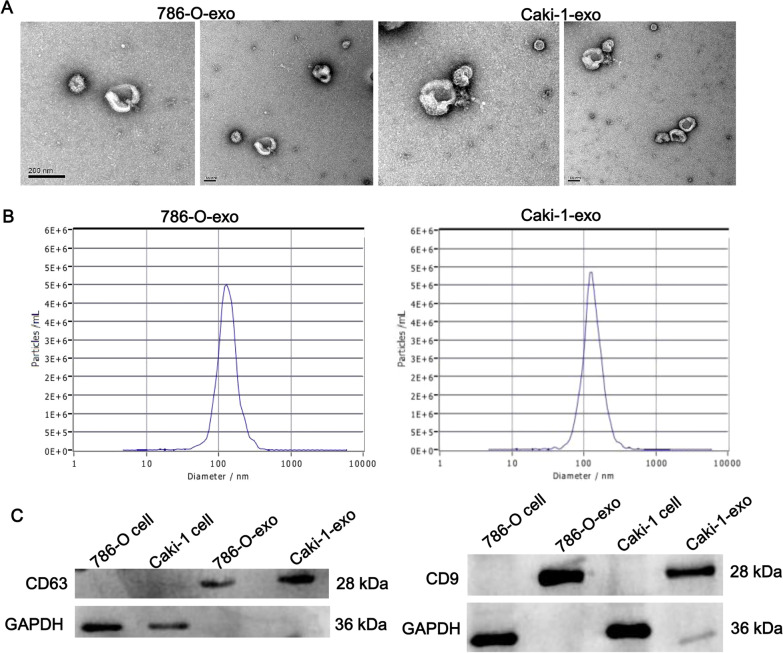
Characterization of exosome derived from ccRCC cells. **A** Transmission electron microscope image of exosomes isolated from 786-O and Caki-1. Scale bar, 100 nm. **B** Nanosight assay showed the size of exosomes distributes to 100 nm. **C** Western Blot detected CD63 and CD9 proteins in cells and exosomes isolated from the cell culture supernatant. GAPDH was detected as a negative control

To investigate the effect of ccRCC-derived exosomes on the macrophage, we extracted exosomes from ccRCC cell lines (786-O and Caki-1). As shown in Fig. 1A, the harvested exosomes characterized typical rounded depression particles with a diameter of about 100 nm. The results of NTA showed that the concentration of the isolated particles was mainly ranging from 30 to 150 nm (Fig. 1B). The exosomes markers, CD63 and CD9 were highly expressed in isolated particles and rarely in cells, confirmed by western blot (Fig. 1C). Therefore, these results suggested that we successfully isolated ccRCC-exo from 786-O and Caki-1 cell lines.

### Exosomes derived from ccRCC promote M2 macrophage polarization

**Fig. 2:**
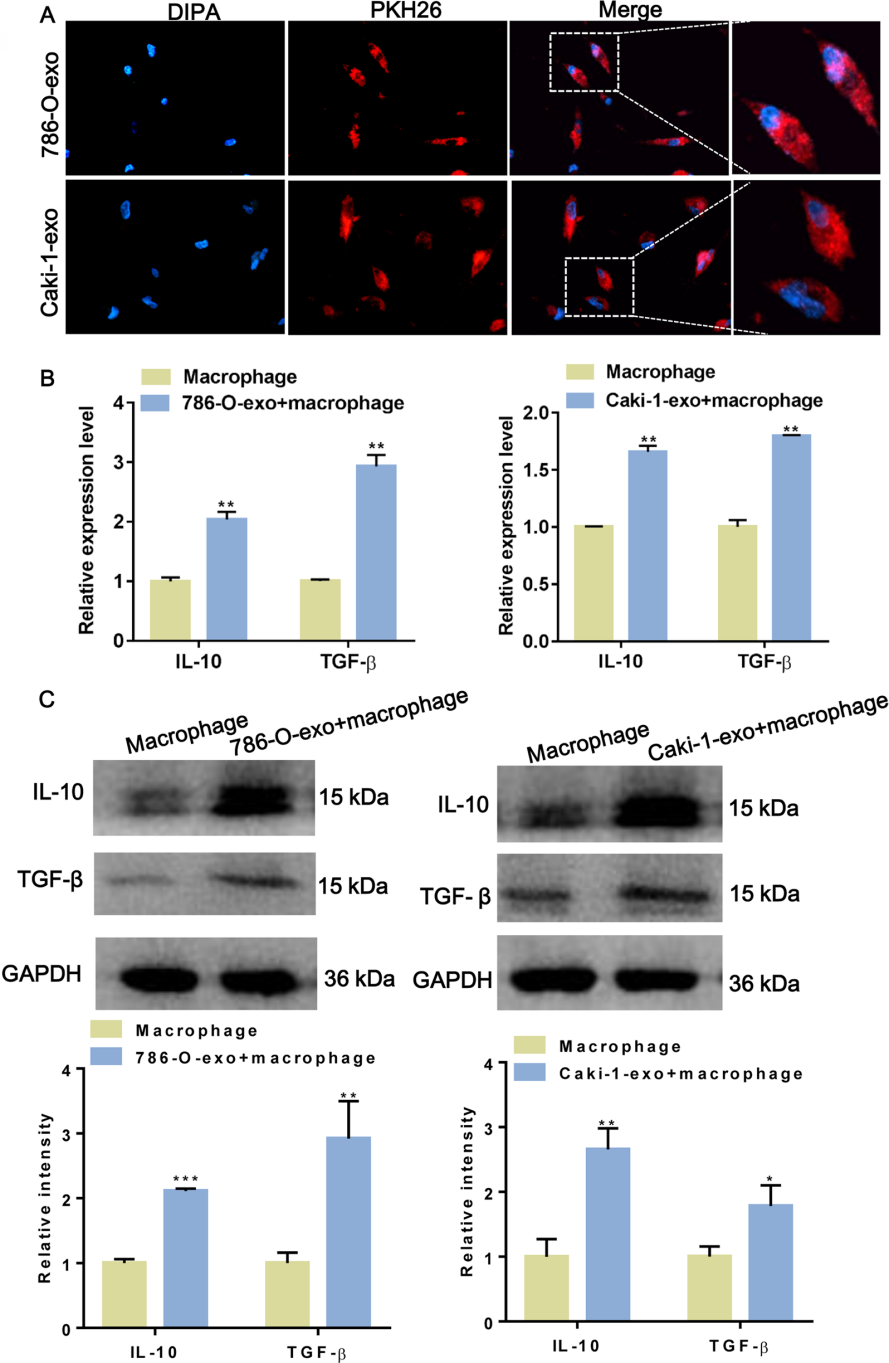
Exosomes derived from ccRCC promoted M2 macrophage polarization. **A** Fluorescence microscopy images proved intercellular trafficking of exosomes labelled with PKH26 dye (magnification, ×200). **B** qRT-PCR was used to detect the mRNA levels of TGF-β and IL-10 in macrophage treated with exosomes derived from 786-O (left) and Caki-1 (right). **C** The protein levels of TGF-β and IL-10 in macrophage were detected by Western Blot. The gray intensities of TGF-β and IL-10 were quantified and standardized with GAPDH. These experiments were done with three distinct biological replicates. *p < 0.05, **p < 0.01, ***p < 0.001. Two-tailed Student’s t-test

Subsequently, we examined whether exosomes derived from ccRCC cells (786-O and Caki-1) affect M2 macrophage polarization. Firstly, we examine whether ccRCC-derived exosomes could be internalized by macrophages. As a result, the PKH26-labeled ccRCC-derived exosomes were internalized by macrophages after co-incubation (Fig. 2A). Next, the effect of ccRCC-derived exosomes on the polarization of macrophages was revealed. The marker of M2 macrophage TGF-β and IL-10 were investigated to increase after co-incubation with 786-O-exo and Caki-1-exo both at mRNA and protein levels (Fig. 2B, C), compared with macrophage culture alone. Therefore, these results indicated that ccRCC-derived exosomes induced M2 macrophage polarization.

### M2 macrophages induced by ccRCC-derived exosomes promote the migration of ccRCC

**Fig. 3:**
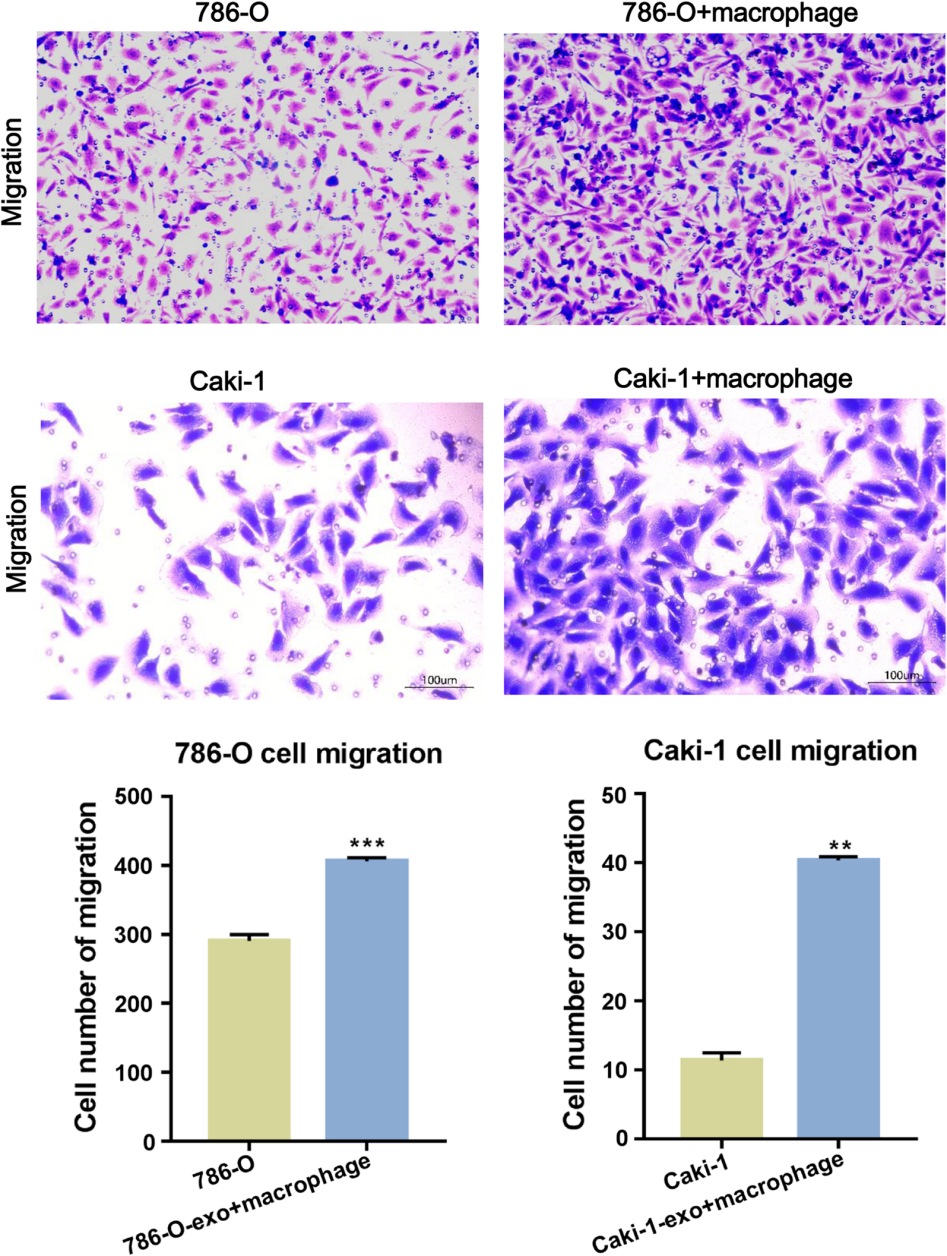
M2 polarized macrophage induced by exosomes from ccRCC promoted the migration of ccRCC. The migration abilities of ccRCC treated with cell culture of macrophage were assessed by transwell experiment. The migrated cells were stained with crystal violet and observed by light microscopy (magnification, × 200). Data represents at least three experiments performed in triplicate. **p < 0.01, ***p < 0.001. Two-tailed Student’s t-test

The previous study demonstrated tumor-derived exosomes can polarize pro-tumorigenic tumor-associated macrophages to support tumor progression [10]. Thus, the effect of ccRCC-derived exosomes-treated M2 macrophages on ccRCC cell migration was determined. Caki-1- and 786-O-derived exosomes-treated M2 macrophages were cocultured with Caki-1 and 786-O cells, and then Transwell system was used to assess the migration ability of cancer cells. As shown in Fig. 3, the migration ability of 786-O and Caki-1 cells were significantly enhanced after co-cultured with M2 macrophages, suggesting that M2 macrophages induced by ccRCC-derived exosomes promote the migration of ccRCC cells.

### Exosomes promote M2 macrophage polarization by carrying lncRNA AP000439.2

**Fig. 4:**
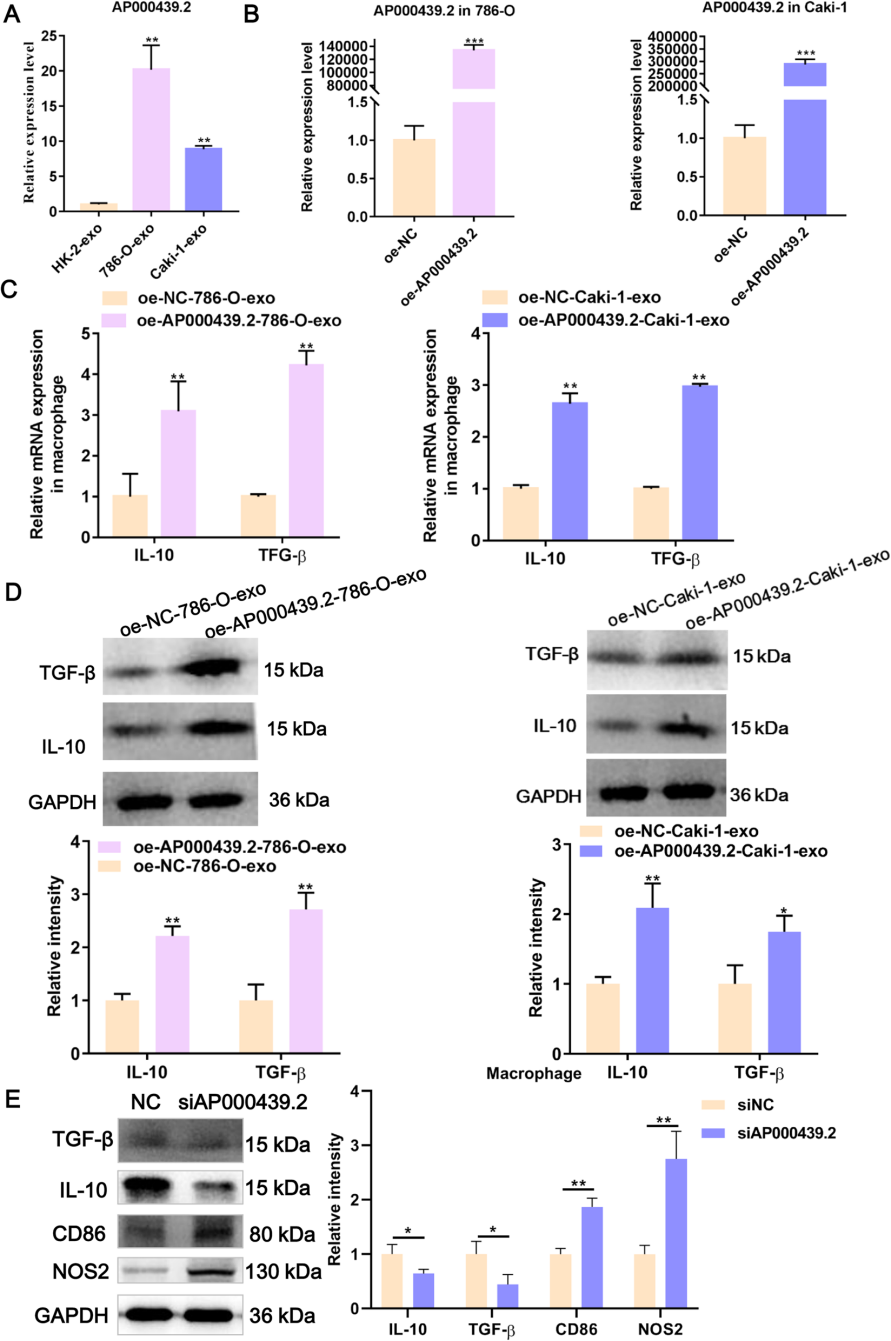
Overexpression of AP000439.2 promotes the M2 polarization of macrophage. **A** The relative expression levels of AP000439.2 in exosomes derived from ccRCC (786-O, Caki-1) and human normal kidney proximal tubular cell line HK-2 detected by qRT-PCR. **B** AP000439.2 expression in AP000439.2 transient overexpressed 786-O and Caki-1 cells detected by qRT-PCR. **C** The mRNA expression level of TGF-β and IL-10 in macrophage was detected by qRT-PCR after co-cultured with exosome. Exosomes were extracted from the cell culture of 786-O and Caki-1 overexpressed AP000439.2, and vector negative control (NC), respectively. **D** The protein expression level of TGF-β and IL-10 in macrophage was detected by western blot after co-cultured with exosome. Exosomes were extracted from the cell culture of 786-O and Caki-1 overexpressed AP000439.2, and NC, respectively. **E** M1 markers (CD86 and NOS2) and M2 markers (TGF-β and IL-10) were detected by western blot in M2 macrophages with AP000439.2 knockdown. Data were analyzed from three distinct biological replicates. *p < 0.05, **p < 0.01, ***p < 0.001, Two-tailed Student’s t-test

**Fig. 5:**
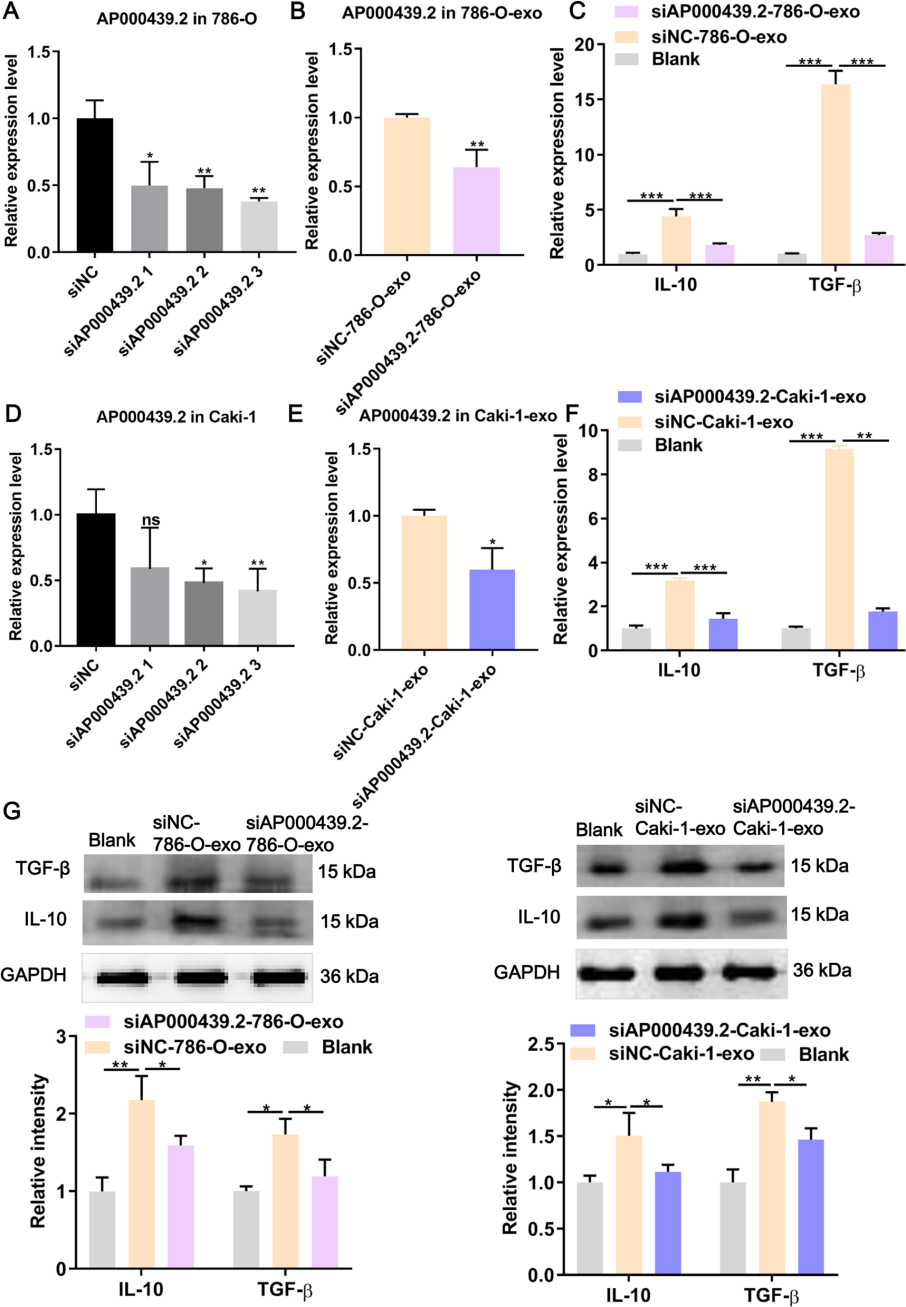
Knockdown of AP000439.2 inhibits the M2 polarization of macrophage. The expression of AP000439.2 in 786-O cells (**A**) and Caki-1 cells (**D**) were knockdown by using siRNA and knockdown efficiency was detected by qRT-PCR. The expression of AP000439.2 in exosomes derived from 786-O cells (**B**) and Caki-1 cells (**E**) after knockdown of AP000439.2 detected by qRT-PCR. The mRNA expression level of TGF-β and IL-10 in macrophage was detected by qRT-PCR after co-cultured with exosome derived from 786-O cells (**C**) and Caki-1 cells (**F**) after knockdown AP000439.2, and NC. (**G**) The protein expression level of TGF-β and IL-10 in macrophage was detected by western blot after co-cultured with exosome derived from 786-O cells and Caki-1 cells after knockdown AP000439.2. Data were analyzed from three distinct biological replicates. *p < 0.05, **p < 0.01, ***p < 0.001, Two-tailed Student’s t-test for two groups, one-way ANOVA following Tukey’s test for three groups

Next, we focus on the molecular mechanism by which ccRCC-derived exosomes promote M2 macrophage polarization. LncRNAs can assume the interaction between tumor exosomes and macrophage polarization in cancer development [16]. In our previous study, we built a classifier based on four lncRNAs that could be promising prognostic tool in predicting the survival of ccRCC patients [17]. In the present study, of the four lncRNAs, only AP000439.2 indicated significantly up-regulated both in exosomes derived from 786-O and Caki-1 cells, compared with that from normal renal cell HK-2 (Fig. 4A). Furthermore, we harvested 786-O- and Caki-1-derived exosomes with overexpressed AP000439.2 (Fig. 4B) and then co-incubated with macrophages, and found that the expression of TGF-β and IL-10 in macrophages was significantly up-regulated at both mRNA and protein levels (Fig. 4C, D). In contrast, AP000439.2-depleted exosomes derived from786-O and Caki-1 cells were significantly down-regulated the mRNA and protein expression of TGF-β and IL-10 in macrophages, compared with siNC group (Fig. 5). Collectively, ccRCC-derived exosomes promoting M2 macrophage polarization was mediated by carrying lncRNA AP000439.2.

To explore whether the effect of AP000439.2 on macrophages is due to macrophage repolarization or to suppression of M2 macrophages, we perforemed experiments in pretreated M2-polarized macrophages. After transfection the siAP000439.2 into M2-polarized macrophages, the M1 markers (CD86 and NOS2) and M2 markers (IL-10 and TGF-β) were detected. The results showed that siAP000439.2 significantly reduced the expression of M2 markers but enhanced the expression of M1 markers in M2-polarized macrophages (Fig. 4E). Therefore, we concluded that siAP000439.2 could repolarized M2 macrophages into M1 phenotype.

### AP000439.2 promote STAT3 phosphorylation in macrophages

**Fig. 6:**
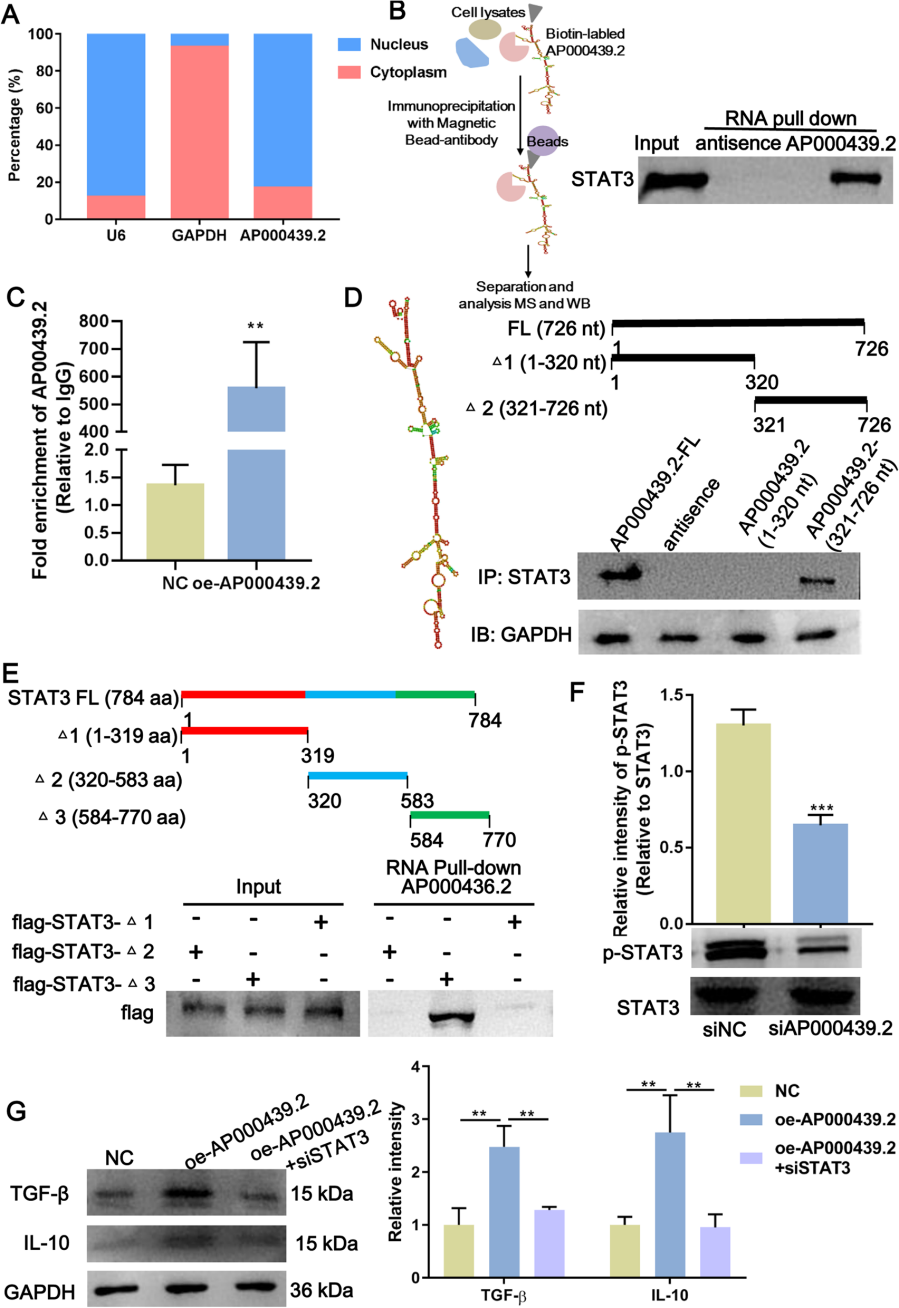
AP000439.2 interacts with STAT3 and phosphorylates STAT3 in macrophage. **A** AP000439.2 was located dominantly in the nucleus proved by cell fractions isolation followed with qRT-PCR. GAPDH was set as a cytoplasm control, and U6 was set as a nucleus control. **B** STAT3 was detected in the antisense and AP000439.2 RNA pull-down sample by western blot. RNA pull-down was performed in macrophages. Antisense RNA probe was used as negative control for AP000439.2 probe. **C** RNA immunoprecipitation with an anti-STAT3 antibody was used to assess whether STAT3 binding to AP000439.2 in macrophages. IgG antibody was used as the control. **D** Deletion mapping of the AP000439.2-binding domain in STAT3. Left, predictive secondary structure of AP000439.2; Up, diagrams of full-length AP000439.2 and the deletion fragments; Bottom, western blot analysis for STAT3 antibody in samples pulled down by the different AP000439.2 constructs. **E** Deletion mapping of the STAT3-binding domain in AP000439.2. Up, diagrams of full-length STAT3 and the deletion fragments; Bottom, western blot analysis for AP000439.2 probe in samples pulled down by the different STAT3 constructs. **F** The phosphorylation level of STAT3 in macrophages decreased upon AP000439.2 knocking down compared with the negative control. **G** The protein expression level of TGF-β and IL-10 in macrophage was detected by western blot after overexpression of AP000439.2 and knockdown of STAT3. **p < 0.01, ***p < 0.001, Two-tailed Student’s t-test for two groups, one-way ANOVA following Tukey’s test for three groups

To further reveal the mechanism of AP000439.2 participating in M2 macrophage polarization, we analyzed the subcellular location of AP000439.2 by qRT-PCR analysis of cell fractions. The results confirmed that AP000439.2 was predominantly distributed in the nucleus and not the cytoplasm (Fig. 6A). Recent studies have shown that lncRNA is involved in the regulation of molecules by interacting with protein [18]. We hypothesized that AP000439.2 may regulate M2 macrophage polarization through binding proteins in the nucleus, thereby we performed AP000439.2 RNA pull-down in macrophage. The co-precipitated proteins were analyzed by mass spectrometry and western blot, we found that STAT3 was the most abundance protein in AP000439.2-protein complex (Fig. 6B), and STAT3 was only detected in the anti-AP000439.2 group not in the antisense group (Fig. 6B). Moreover, RIP assay confirmed that AP000439.2 could be enrichment by STAT3 antibody in contrast to IgG antibody, and overexpression of AP000439.2 significantly enhanced AP000439.2 accumulation when against anti-STAT3 (Fig. 6C). These results indicated that AP000439.2 bound specifically to STAT3 in macrophage cells. To determine the region of AP000439.2 to which STAT3 binds, we prepared two AP000439.2 truncation mutants according to its predictive secondary structure and found that 321–726 nt of AP000439.2 was sufficient to bind STAT3 (Fig. 6D). Similarly, we tested which domain of STAT3 proteins is required for binding to 321–726 nt of AP000439.2. We prepared three STAT3 truncation mutants and found that ^△^3 truncation mutants of STAT3 still interacted with AP000439.2, while other STAT3 protein truncated mutants could not (Fig. 6E), suggesting that 584–770 aa region of STAT3 was required for binding AP000439.2. Furthermore, we found that knocking down AP000439.2 did not alter STAT3 expression but significantly decreased the phosphorylation of STAT3 in macrophages (Fig. 6F). Importantly, silencing STAT3 suppressed the overexpression of AP000439.2-induced up-regulation of TGF-β and IL-10 expression (Fig. 6G). Taken together, AP000439.2 promote phosphorylation of STAT3 through specifically interaction in macrophage which contributing to facilitate M2 macrophage polarization.

### AP000439.2 activating NF-κB signaling pathway via phosphorylated STAT3

**Fig. 7:**
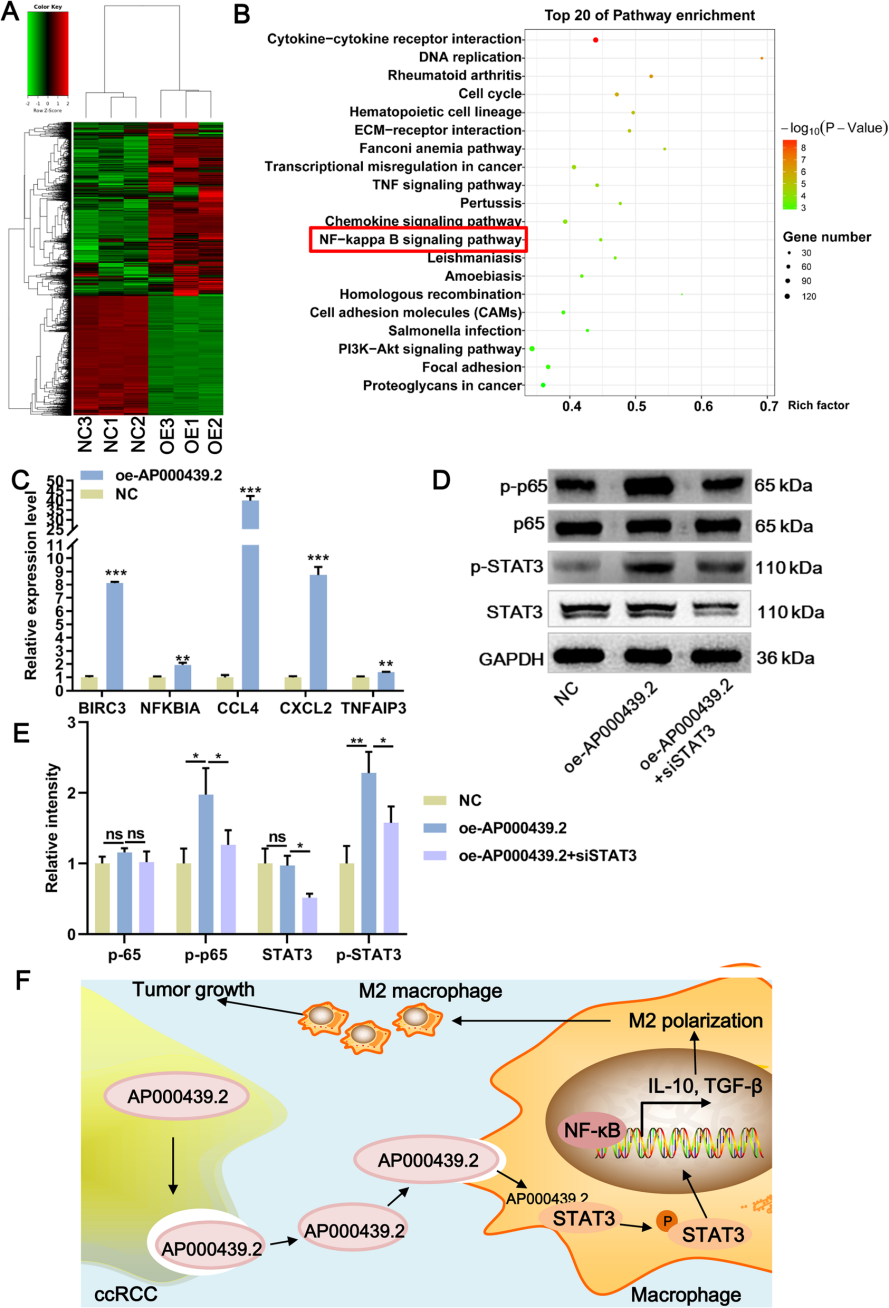
AP000439.2 interacts with STAT3 and regulates the NF-κB pathway in macrophage. **A** Heat map of differentially expressed genes in macrophage pretreated with AP000439.2-overexpressed exosome or NC-exo. OE1, OE2 and OE3 represent AP000439.2-overexpressed exosome group. C1, C2, and C3 represent the control group. **B** Top 20 KEGG enrichment pathways of differentially expressed genes. **C** The expression of 5 differentially expressed genes in NF-κB signaling pathway was verified by qRT-PCR. **D** The phosphorylation level of p65 (a representative gene for activation of NF-κB signaling pathway) in macrophages after overexpression of AP000439.2 and knockdown of STAT3. **E** Statistical analysis results of western blot. **F** A graphical summary of lncRNA AP000439.2 regulation. Exosomal lncRNA AP000439.2 derived from ccRCC promote the M2 macrophage polarization though phosphorylated STAT3 and activated NF-κB signaling pathway, thus facilitate the progression of ccRCC. ns p > 0.05, *p < 0.05, **p < 0.01, ***p < 0.001, Two-tailed Student’s t-test for two groups, one-way ANOVA following Tukey’s test for three groups

Subsequently, RNA-Seq was performed to identify DEGs and signaling pathways in macrophages in response to AP000439.2 overexpression. A total of 3621 up-regulated DEGs and 2496 down-regulated DEGs were identified in AP000439.2-overexpressed exosome-treated macrophage, compared with NC group (Fig. 7A). Kyoto Encyclopedia of Genes and Genomes (KEGG) enrichment analysis revealing that AP000439.2 overexpression activated multiple macrophage polarization-related pathways, such as NF-κB signaling pathway, and PI3K-Akt signaling pathway (Fig. 7B). It has been reported that increased STAT3 phosphorylation by IL-6 mediated M2 macrophages polarization [19]. Moreover, STAT3 could activate of NF-κB signaling pathway through mediating p100 process to p52, and this way was perceived to be a common pathway of representing cancer cells to survive and escape therapy [20]. NF-κB signaling pathway have been shown to promote production of varies polarization cytokines such as TNF-α, IL-10, IL-4, and TGF-β [21–23]. Therefore, we hypothesized that AP000439.2 binds with and phosphorylates STAT3, activating the NF-κB signaling pathway which leading to M2 macrophage polarization. To verify our speculation, we performed a simple qRT-PCR and western blot verification experiment. Five DEGs enriched in the NF-κB signaling pathway were selected for qRT-PCR detection, and their expression were significantly upregulated in AP000439.2-overexpressed exosome-treated macrophage compared with NC (Fig. 7C). We also measured phosphorylation of p65, a representative gene for activation of NF-κB signaling pathway. As shown in Fig. 7D, compared with NC group, AP000439.2 overexpression increased the NF-κB p65 phosphorylation while this increase was significantly reduced by knockdown of STAT3. But overexpression AP000439.2 and knockdown of STAT3 have no effect on p65 expression. Moreover, consistent with Fig. 6F, ectopic expression of AP000439.2 only affect the phosphorylation of STAT3 but not the protein expression (Fig. 7D, E). These results indicated that AP000439.2 phosphorylated STAT3 by interaction to activate NF-κB signaling pathway resulting in macrophages M2 polarization, as the ccRCC eventually progressed (Fig. 7F).

### AP000439.2-deleted exosome suppressed tumor growth

**Fig. 8:**
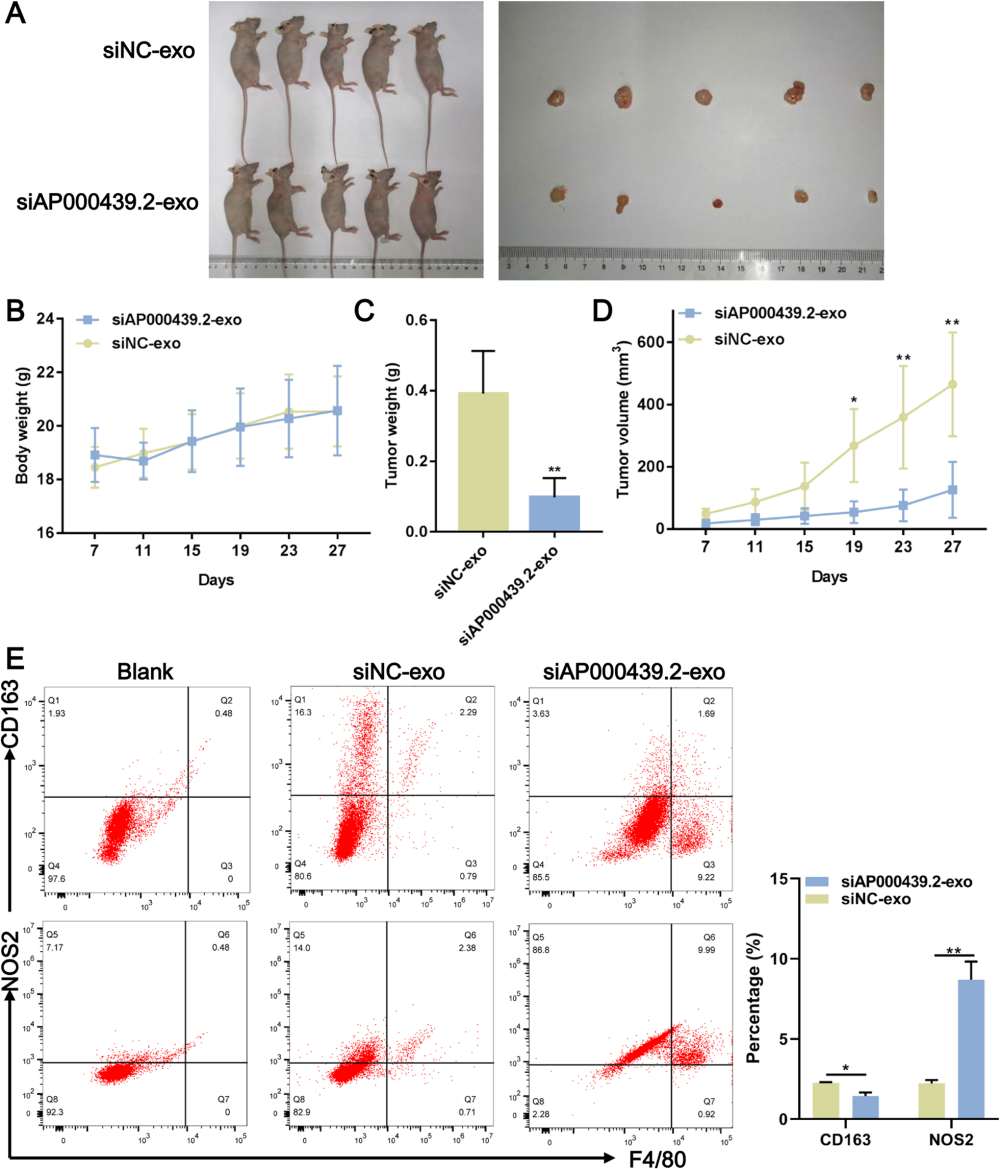
AP000439.2-deleted exosome suppressed tumor growth. Xenograft tumor was established by injecting Caki-1 cells, in the meantime, mice treated with AP000439.2-deleted exosome or NC exosome via tail vein injection. **A** Xenograft tumor images from each mouse. **B** Body weight curve of mice. **C** Tumor weight of mice from two groups. **D** Tumor volume curve of mice from two groups. **E** The proportion of M1 (F4/80^+^NOS2^+^) and M2 (F4/80^+^CD163^+^) macrophages in tumor tissues by flow cytometry. *p < 0.05, **p < 0.01. Two-tailed Student’s t-test

To further evaluate the therapeutic efficacy of AP000439.2-deleted exosome in ccRCC in vivo, a ccRCC xenograft tumor model was established using Caki-1 cell line (Fig. 8A). We found that AP000439.2-deleted exosome treatment had no effect on body weight in mice (Fig. 8B), but significantly reduced tumor weight (Fig. 8C), compared with siNC-exo group. The tumor growth curve showed that a significant therapeutic effect of AP000439.2-deleted exosome was registered as from 19 days of tumor-burden onwards (Fig. 8D). The above results indicated that AP000439.2-deleted exosome can be used in vivo to suppress ccRCC growth. Furthermore, to determine whether the reduction in tumor growth results from repolarization of tumor-associated macrophages or suppression of M2 macrophages, we quantified the proportion of M1 and M2 macrophages in tumor tissues by flow cytometry. Compared with the siNC-exo group, the positive proportion of CD163 was significantly decreased in siAP000439.2-exo group, while the NOS2 was significantly increased (Fig. 8E), suggesting that siAP000439.2-exo promoted the proportion of M1 macrophage and reduced proportion of M2 macrophage. Therefore, consistent with that in vitro, the reduction in tumor growth by siAP000439.2-exo results from repolarization.

## Discussion

TAM with different polarization phenotypes plays different roles in tumor genesis, metastasis, and relapse [24, 25]. According to the report, a large infiltration of M2 macrophage in tumors indicated a poor prognosis in non-small-cell lung cancer [26]. Increasing evidence suggests exosomes from tumor cells has emerged as a critical regulatory circuit to communicate with TAM to contribute to tumor progression. However, there are no reports on whether the ccRCC-derived exosomes affect TAMs polarization and tumorigenesis. Here, our founding reveals that exosomal lncRNA AP000439.2 derived from ccRCC promote the M2 macrophage polarization though phosphorylated STAT3 and activated NF-κB signaling pathway, thus facilitate the progression of ccRCC in vitro and in vivo (Fig. 7E).

Our results show that the expression of lncRNA AP000439.2 in ccRCC-derived exosomes was significantly higher than that from normal cells, and overexpression of AP000439.2 promote M2 macrophage polarization. To our knowledge, study characterizing AP000439.2 function is very limited. AP000439.2 was screened out from RNA-Seq data and was considered a prognostic biomarker for ccRCC in our previous study [17]. AP000439.2 was also presumed as a clinical marker in lung adenocarcinoma on the basis of bioinformatic analysis [27]. However, experimental verification and mechanism studies have not yet been reported. We found that AP000439.2-overexpressed exosome promoted tumorigenic polarization, while AP000439.2-knockout exosomes did the opposite effect. This study for the first time experimentally confirmed the role of AP000439.2 in guiding tumorigenic polarization of TAM. Our results provides a reference for making macrophage plasticity an attractive target for therapeutic interventions through exosomal AP000439.2.

In the present study, the results reveal that exosomal AP000439.2 promotes M2 polarization by phosphorylating STAT3 through interaction to regulating NF-κB signaling pathways in macrophages. The STATs family and NF-κB have been reported to play a core role in the activation of M2 macrophage polarization [9, 28]. For example, blocking STAT3 phosphorylation prevents immune evasion exerted by supraglottic laryngeal tumor induced M2 macrophages [29]. Suppression of STAT6 phosphorylation by knockdown of lncRNA small nucleolar RNA host gene 1 (SNHG1) inhibited M2 macrophage polarization in breast cancer [30]. Study reported that lncRNA nuclear-enriched abundant transcript 1(NEAT1) sponged miR-214 to target regulation B7-H3 thereby promoted M2 macrophage polarization via phosphorylating JAK2/STAT3 signaling in multiple myeloma [31]. Consistent with these results, we found that AP000439.2 delivering by ccRCC-derived exosomes promoted M2 macrophage polarization via phosphorylated STAT3. Therefore, exosomal AP000439.2 impose M2 macrophage polarization through interacting and phosphorylating STAT3. In addition, NF-κB signaling pathway also serves as an important factor for activating M2 macrophages polarization. For instance, lncRNA DC-STAMP domain-containing 1-antisense 1 (DCST1-AS1) supports oral squamous cell carcinoma progression via facilitating M2 macrophage polarization through activating NF-κB signaling [32]. An et al. reported that lncRNA SNHG16 promoted proliferation and M2 macrophages polarization, characterized by the increasing levels of TNF-α, IL-1β and IL-6, through miR-17-5p/NF-κB signaling pathway in atherosclerosis patients [33]. Consistent with these results, our transcriptome sequencing confirmed that AP000439.2 overexpression led to NF-κB signaling activation. Taken together, exosomal AP000439.2 interacting STAT3 facilitated NF-κB signaling activation which imposed M2 macrophage polarization, eventually accelerated ccRCC migration.

## Conclusion

In summary, ccRCC-derived exosomes promote M2 macrophage polarization thereby contribute to tumor progression via transport lncRNA AP000439.2. Furthermore, exosomal LncRNA AP000439.2 interacts with and phosphorylates STAT3 to activate NF-κB signaling pathway resulting in M2 macrophage polarization. A novel therapeutic target of AP000439.2 has been paved for ccRCC.

## Supplementary Information

**Additional file 1**. **Table S1**: The primers used in this study.

## Data Availability

The datasets used and/or analysed during the current study are available from the corresponding author on reasonable request.

## Abbreviations

TAMs: Tumor-associated macrophages
RCC: Renal cell carcinoma
ccRCC: Clear cell renal cell carcinoma
ELISA: Enzyme-linked immunosorbent assay
RCC: Renal cell carcinoma
TME: Tumor microenvironment
lncRNAs: Long noncoding RNAs
TEM: Transmission electron microscopy
STAT3: Signal transducer and activator of transcription 3
DEGs: Differentially expressed genes
FBS: Fetal bovine serum
PBS: Phosphate buffer saline
TGF: Transforming growth factor
IL: Interleukin
CXCL17: C-X-C motif chemokine ligand 17
BCRT1: Breast cancer-related transcript 1
PTBP3: Polypyrimidine tract binding protein 3
NTA: Nanoparticle tracking analysis
DAPI: 4,6-Diamidino-2-phenylindole
TBST: Tris buffered saline Tween-20
RIP: RNA immunoprecipitation
KEGG: Kyoto Encyclopedia of Genes and Genomes
SNHG: Small nucleolar RNA host gene
NEAT1: Nuclear-enriched abundant transcript 1
DCST1-AS1: DC-STAMP domain-containing 1-antisense 1
