# Supplementary material for: Exosomal AP000439.2 from clear cell renal cell carcinoma induces M2 macrophage polarization to promote tumor progression through activation of STAT3
Source: Cell Commun Signal. 2022 Sep 24;20:152. doi: 10.1186/s12964-022-00957-6 (PMC9509597; doi:10.1186/s12964-022-00957-6)
Supplement: Supplementary file 2 — Additional file 1. Table S1: The primers used in this study. [file 12964_2022_957_MOESM2_ESM.docx]

Table S1. The primers used in this study.

| Gene | Primers (5’ - 3’) |
| --- | --- |
| Actin-F | AGCACAGAGCCTCGCCTTTG |
| Actin-R | CTTCTGACCCATGCCCACCA |
| TGF-β-F | CCTGGCGATACCTCAGCAAC |
| TGF-β-R | CTAAGGCGAAAGCCCTCAAT |
| IL10-F | TGCCAAGCCTTGTCTGAGATG |
| IL10-R | AGATGTCAAACTCACTCATGGCTT |
| GAPDH-F | AGAAGGCTGGGGCTCATT |
| GAPDH-R | TGCTAAGCAGTTGGTGGTG |
| AP000439.2-F | TACTGGGCTAGGCGTCAGAT |
| AP000439.2-R | GATGGCCCAGATACATCGCA |
| siSTAT3 | GCAACAGAUUGCCUGCAUUTT |
|  | AAUGCAGGCAAUCUGUUGCTT |
| siAP000439.2 1 | ACAUCUUCCUAGCCUGGGGGG |
|  | CCCCAGGCUAGGAAGAUGUGC |
| siAP000439.2 2 | AUUCUCAAUGCCUUUCAACCU |
|  | GUUGAAAGGCAUUGAGAAUCU |
| siAP000439.2 3 | UUCUUCACCCCGAACUUCCCC |
|  | GGAAGUUCGGGGUGAAGAAGG |

Note: F means forward primers, R means reverse primers.
